# New Insights into the Association between Fibrinogen and Coronary Atherosclerotic Plaque Vulnerability: An Intravascular Optical Coherence Tomography Study

**DOI:** 10.1155/2019/8563717

**Published:** 2019-04-02

**Authors:** Jun Wang, Lu Jia, Xing Li, Siyu Jin, Xiaomei Li, Fen Liu, Chunfang Shan, Yu Zhang, Yining Yang

**Affiliations:** Department of Coronary Heart Disease, the First Affiliated Hospital of Xinjiang Medical University, Urumqi 830011, China

## Abstract

**Background:**

Fibrinogen levels have been associated with coronary plaque vulnerability in experimental studies. However, it has yet to be determined if serum fibrinogen levels are independently associated with coronary plaque vulnerability as detected by optical coherence tomography (OCT) in patients with coronary heart disease.

**Methods:**

Patients with coronary heart disease (CHD) who underwent coronary angiography and OCT in our department from January 2015 to August 2018 were included in this study. Coronary lesions were categorized as ruptured plaque, nonruptured with thin-cap fibroatheroma (TCFA), and nonruptured and non-TCFA. Presence of ruptured plaque and nonruptured with TCFA was considered to be vulnerable lesions. Determinants of coronary vulnerability were evaluated by multivariable logistic regression analyses.

**Results:**

A total of 154 patients were included in this study; 17 patients had ruptured plaques, 15 had nonruptured plaques with TCFA, and 122 had nonruptured plaques with non-TCFA. Results of univariate analyses showed that being male, diabetes, current smoking, high body mass index (BMI), and clinical diagnosis of acute coronary syndrome (ACS) were associated with coronary vulnerability. No significant differences were detected in patient characteristics, coronary angiographic findings, and OCT results between patients with higher and normal fibrinogen. Results of multivariate logistic analyses showed that diabetes and ACS were associated with TCFA, while diabetes, higher BMI, and ACS were associated with plaque rupture.

**Conclusions:**

Diabetes, higher BMI, and ACS are independently associated with coronary vulnerability as detected by OCT. Serum fibrinogen was not associated with coronary vulnerability in our cohort.

## 1. Introduction

Conventional cardiovascular risk factors, such as smoking, diabetes, hypertension, and dyslipidemia, have been associated with incidence of acute cardiovascular adverse events in patients with coronary heart disease (CHD) [1]. However, acute coronary events can occur in patients without conventional cardiovascular risk factors, indicating the presence of unknown risk factors [1, 2]. Pathologically, incidences of acute coronary events have been related to coronary lesion vulnerability [3]. Therefore, identifying novel factors associated with coronary plaque vulnerability may be important for predicting acute coronary events in CHD patients. Accumulating evidence suggests that plasma fibrinogen, an active factor involved in coagulation, may contribute to the risk of acute thrombotic disease via its proinflammatory effects [4]. Elevated fibrinogen levels have been observed in patients who are at higher risk for CHD, such as those who smoke and have diabetes, hypertension, obesity, lipid metabolism disorders, menopause, and depression [5, 6]. In contrast, factors that reduce CHD risk, such as regular exercise, also reduce fibrinogen levels [7, 8]. Experimental studies have also suggested that fibrinogen and fibrin degradation products may increase coronary plaque vulnerability by stimulating coagulation, platelet aggregation, and vascular endothelial dysfunction [9]. Clinical studies have also demonstrated that fibrinogen is correlated with atherosclerosis severity, as determined by both coronary angiography (CAG) and carotid ultrasonography [10, 11]. However, whether plasma fibrinogen is independently associated with coronary lesion vulnerability in CHD patients remains to be determined.

Optical coherence tomography (OCT) is an emerging tool used to evaluate coronary plaque vulnerability* in vivo*. OCT can provide intraluminal evidence that confers more accurate findings of plaque characteristics compared to intravascular ultrasound (IVUS) imaging [12]. Although the association between fibrinogen and* in vivo* coronary plaque characteristics has only been examined using IVUS [13, 14], the literature does not provide any evidence that plasma fibrinogen is independently associated with coronary lesion vulnerability as detected by OCT. The aim of the current study was to evaluate the potential association between fibrinogen and coronary vulnerability using OCT.

## 2. Methods

### 2.1. Patient Population

**Figure 1:**
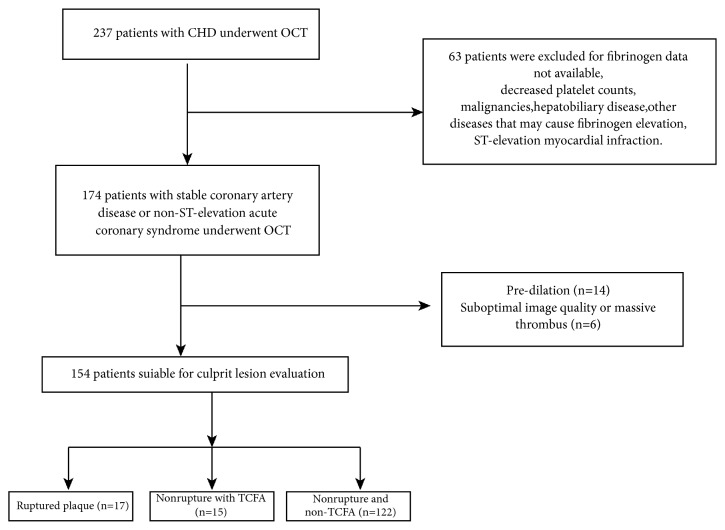
Flowchart of patient enrollment.

Patients with CHD who were scheduled to receive coronary angiography and OCT in our department from January 2015 to August 2018 were included in this study. Patients with either stable coronary artery disease (SAP) or non-ST-elevation acute coronary syndrome NSTE-ACS were eligible for study inclusion. Diagnosis was in accordance with previously established guidelines [15]. The flow chart for patient inclusion and exclusion is shown in Figure 1. Patients with the following clinical conditions were excluded, as these factors may affect fibrinogen plasma levels: decreased white blood cell counts, decreased platelet counts, hepatic or renal dysfunction, inflammatory disease, prolonged occluded coronary bypass graft, malignant tumors, and other diseases that may cause fibrinogen elevation. Written informed consent for CAG and OCT were obtained from all patients. The study protocol was approved by the local ethics committee.

### 2.2. Definition of Cardiovascular Risk Factors

Hypertension was defined as elevated blood pressure, including systolic blood pressure (SBP) > than 140 mmHg or diastolic blood pressure (DBP) > than 90 mmHg. Patients with a reported history of hypertension and who had used any antihypertensive medications were also considered hypertensive [16]. Dyslipidemia was defined using current guidelines [17]: low-density lipoprotein cholesterol (LDL-C) > 3.1 mmol/L, triglyceride (TG) > 2.3, mmol/L, high-density lipoprotein cholesterol (HDL-C) < 1.0, mmol/L, and total cholesterol (TC) > 5.2 mmol/L. A lipoprotein (a) (Lp(a)) > 300 mg/L has also been listed as a risk factor for cardiovascular diseases [18, 19]. Body mass index (BMI) was determined by ratio of body weight (kg) to height (m^2^). A BMI > 28 kg/m^2^ was considered obesity, and BMI between 24 – 28 kg/m^2^ was considered overweight [20]. Diabetes mellitus (DM) was diagnosed when glucose > 126 mg/dL or glycated hemoglobin (HbA1c) was > 6.5%, in the presence of active treatment with insulin or oral antidiabetic agents, in accordance with the American Diabetes Association criteria [21].

### 2.3. Blood Tests

Blood samples were collected from patients in the fasting state. Serum samples were separated by centrifugation, stored at 4°C, and then analyzed (Dimension AR/AVL Clinical Chemistry System, Newark, NJ, USA). Lipid profile, coagulation function, and other routine blood biochemical parameters were obtained.

### 2.4. Coronary Angiography and OCT Analyses

**Figure 2:**
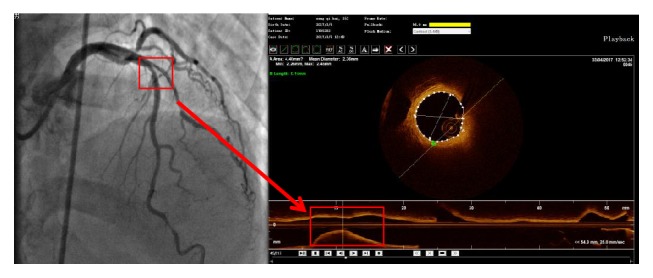
Representative images of lesion plaques analyzed by optical coherence tomography.

**Figure 3:**
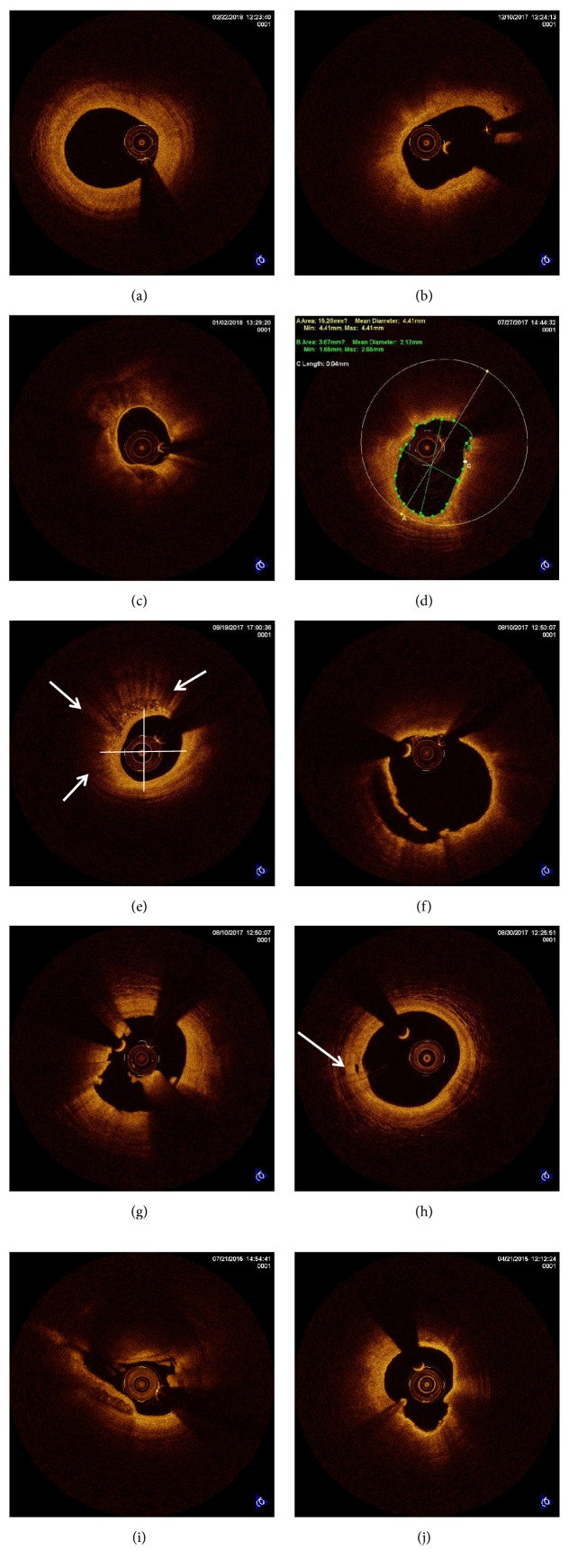
Representative optical coherence tomography (OCT) images of coronary atherosclerotic plaques with different characteristics. (a) Fibrotic plaque is characterized by a homogeneous OCT signal and high backscattering. (b) A fibroatheroma was characterized by an atherosclerotic plaque with an OCT-delineated necrotic core (formed by a signal-poor region with poorly delineated borders and little or no OCT backscattering), covered by a fibrous cap (signal-rich layer). (c) A calcific fibroatheroma was characterized by a plaque containing calcium deposits (signal-poor regions with sharply delineated borders). (d) A thin-cap fibroatheroma was characterized by a plaque with lipid content in ≥ 2 quadrants and with a fibrous cap < 65 *μ*m. (e) Macrophage accumulation was reflected by a signal-rich punctate region in the background of an atherosclerotic plaque. Macrophages could be quantitatively classified as follows: grade 0, no macrophage; grade 1, localized macrophage accumulation; grade 2, clustered accumulation < 1 quadrant; grade 3, clustered accumulation ≥ 1 quadrant but < 3 quadrants; and grade 4, clustered accumulation ≥ 3 quadrants. (f) Plaque rupture was characterized by discontinuity of the fibrous cap with a cavity formed inside the plaque. (g) Intracoronary thrombus was characterized by a mass (diameter > 250 mm) that could be attached to the luminal surface or floating within the lumen. A red thrombus that was rich in red blood cells could be identified by high backscattering and high attenuation, while a white thrombus that was rich in platelets could be identified by homogeneous backscattering with low attenuation. (h) The vasa vasorum was characterized by voids with poor signals that were sharply delineated in multiple contiguous frames. (i) Calcified nodules were characterized by a small nodular calcification protruding from the lumen at the base of the fibrous calcified plaques with thrombus formation. (j) Acute Coronary Syndrome with Intact Fibrous Cap (ACS-IFC) was characterized by the following three conditions: (1) presence of the attached thrombus overlying an intact and visualized plaque; (2) irregularity of the luminal surface at the culprit lesion in the absence of thrombus; or (3) attenuation of the underlying plaque by thrombus that was not near a superficial lipid or calcification.

Coronary angiography was performed for each patient by an experienced cardiologist using a standard procedure. Culprit vessels, defined as the vessels with the most severe lesions, for each patient were analyzed using OCT (C7-XR TM OCT Intravascular Imaging System, St. Jude Medical, St. Paul, MN, USA). OCT images were digitized and analyzed by scanning the culprit vessel using an automatic retraction device (Figure 2). Image-pro Plus analysis software was used to analyze the lesion plaques, including plaque type, fiber cap thickness, macrophage rating, plaque rupture, acute coronary syndrome with intact fibrous cap (ACS-IFC), thrombosis, trophoblast vessels, and calcified nodules (described in detail in Figure 3) [22–24]. All OCT images were analyzed by two independent investigators (J.L and S.C.F) who are hospital senior professional and technical personnel and were blinded to the clinical angiographic and laboratory data. Inconsistencies were solved by consensus with a third investigator.

### 2.5. Statistical Analysis

Continuous data are presented as mean ± standard deviation (SD) or median (interquartile range), and categorical data are presented as numbers and percentages. Between-group differences were tested using an independent sample t-test or the Mann-Whitney U test. Categorical data are presented as counts (proportions) and were compared using the **χ**^2^ test or Fisher's exact test. Multiple logistic regression analyses were performed to assess the independent predictors of plaque rupture (Model 1) and TCFA (Model 2). The parameters that showed statistical significance in univariate analysis were included in the multivariate logistic regression analyses. A two-sided P value < 0.05 was considered statistically significant. All statistical analyses were performed using SPSS Software.

## 3. Results

### 3.1. Coronary Risk Factors and Biochemical Parameters

A total of 154 patients with CHD were included in this study: 95 patients had stable angina pectoris (SAP), 37 had unstable angina pectoris (UAP), and 22 had non-ST-segment-elevation myocardial infarction (NSTEMI). The baseline characteristics of coronary risk factors and biochemical parameters are presented in Table 1. Significant differences were detected for gender, diabetes, smoking, BMI, and ACS diagnosis among the three groups. Patients with ruptured plaque or nonrupture with TCFA were more likely to be male, diabetic, a current smoker, and with ACS compared to those with nonrupture and non-TCFA (P all < 0.05). Moreover, patients with ruptured plaque had higher BMI compared to those with nonrupture with TCFA and nonrupture with non-TCFA. Plasma levels of fibrinogen were not statistically different among the three groups.

### 3.2. Coronary Angiographic Findings and OCT Analysis

Angiographic findings and OCT analysis results are shown in Table 2. Although the primary CAG findings were not significantly different among the three groups, OCT analysis showed considerable differences in minimal fibrous cap thickness, lipid arc, macrophage accumulation, and thrombus formation. Specifically, fiber cap thickness in the plaque rupture group was lower compared to the nonplaque rupture combined with nonplaque rupture with TCFA group (P < 0.001). Lipid arc in the plaque rupture group was higher compared to the nonplaque rupture with TCFA group (P < 0.001). Macrophage accumulation in the plaque rupture group was higher compared to the nonplaque rupture with TCFA group (P < 0.001). The incidence rate of thrombus in the plaque rupture group was higher compared to the nonplaque rupture with TCFA group (P < 0.001). Fiber cap thickness in the nonrupture and nonplaque rupture with TCFA group was lower compared to the nonrupture and non-TCFA group (P < 0.001). The lipid arc of the TCFA group was higher compared to the nonplaque rupture group (P < 0.001). Macrophage accumulation in the TCFA group was higher compared to the nonrupture and non-TCFA group (P < 0.001). The incidence rate of thrombus in the non-TCFA group was higher compared to the nonrupture and non-TCFA group (P < 0.001).

### 3.3. Association between Patient Characteristics and Coronary Vulnerability by OCT

Model 1 indicates the outcomes of the plaque rupture versus the nonplaque rupture with TCFA groups, and Model 2 indicates the outcomes of the nonplaque rupture with TCFA versus the nonrupture and non-TCFA groups. Results of multivariate logistic analyses showed that diabetes (odds ratio (OR): 4.703, P = 0.036), ACS (OR: 4.418, P = 0.037), and higher BMI (OR: 1.572, P = 0.001) were independently associated with plaque rupture, while diabetes and ACS were independently associated with plaque rupture and TCFA (Table 3).

### 3.4. Relationship of Fibrinogen Level with Patient Characteristics and OCT Findings

Fibrinogen levels according to different conventional CHD risk factors, biochemical parameters, and concurrent medications are shown in Table 4. Plasma fibrinogen levels were not significantly affected by the above factors. Moreover, no statistical difference was detected for CAG and OCT findings between patients with normal or higher fibrinogen levels (Table 5).

## 4. Discussion

In this study, we found that plasma fibrinogen levels were not associated with coronary lesion vulnerability as determined using OCT. Moreover, diabetes and ACS were independently associated with coronary lesion vulnerability, as determined by TCFA and plaque rupture in OCT. Similarly, diabetes, ACS, and obesity were independent determinants of plaque rupture in OCT. These findings contrasted the previous hypothesis that higher plasma fibrinogen levels may be a marker or risk factor for coronary lesion vulnerability.

### 4.1. Fibrinogen and Coronary Atherosclerotic Plaque Vulnerability

Plaque rupture and TCFA have been established as manifestations of plaque vulnerability in OCT studies [22]. Both plaque rupture and TCFA are the key pathophysiological features of ACS. However, previous studies suggested that plasma fibrinogen may accelerate the process of plaque rupture via its proinflammatory [25] and prothrombotic [26] effects. Thus, it was proposed that increased plasma fibrinogen levels in CAD patients may serve as a biomarker of atherosclerosis burden [27]. Our study, using the current gold-standard tool to evaluate coronary vulnerability, indicated that fibrinogen levels were not independently associated with OCT derived features of coronary vulnerability, including plaque rupture and TCFA development. However, antiplatelet therapy and statins can influence the detection of vulnerable plaques [28, 29]. In our study, medications were not statistically different among the three groups. These results suggest that the potential association between fibrinogen levels and coronary vulnerability raised in previous studies may be confounded by other CHD risk factors. This is inconsistent with previous studies that showed that fibrinogen was independently associated with coronary severity in CHD patients [30]. Of note, CAG, rather than intraluminal tools, was used to evaluate coronary lesion severity. Interestingly, another study using IVUS showed that fibrinogen levels correlated with plaque progression [13]. However, only 60 patients were included in that study. Similarly, another study using VH-IVUS concluded that fibrinogen degradation products are associated with larger plaques that have a larger necrotic core [14], but this finding was not confirmed by a subsequent large study that also used histology-IVUS. This study also did not confirm a relationship between fibrinogen and TCFA [31]. One explanation for the inconsistent findings is that genetic factors, such as polymorphisms in fibrinogen loci raised by a multiethnic meta-analysis [32], may confound the association between fibrinogen and coronary vulnerability. However, results of our study provide a more accurate association, since OCT yields higher resolution compared to IVUS to evaluate intraluminal lesions in the coronary artery [33]. Although experimental studies have demonstrated multiple mechanisms underlying the potential role of fibrinogen for accelerating coronary plaque vulnerability [34–39], the current findings in CHD patients did not support a significant effect of fibrinogen on coronary vulnerability, which may reflect the complexity of the pathogenesis of plaque rupture.

### 4.2. Diabetes and Coronary Atherosclerotic Plaque Vulnerability

Type 2 diabetes has been established as one of the most important risk factors for CHD [40]. Diabetic patients have greater macrophage infiltration and large necrotic cores in their coronary lesions compared to those without diabetes, which confers an increased risk for acute coronary events [41]. However, previous findings on diabetes and coronary vulnerability were mostly derived from experimental studies. Related studies in CHD patients using OCT to evaluate coronary vulnerability have been rarely reported. Here, we showed that diabetes is independently associated with OCT confirmed coronary vulnerability as presented by TCFA and plaque rupture, which is consistent with previous pathology studies. Moreover, this is consistent with a recent study that showed that high glycemic variability was associated with increased OCT-detected plaque vulnerability in nonculprit lesions [42]. After correcting for other confounders, such as ACS, our results support previous OCT studies demonstrating the differences in TCFA prevalence at the culprit lesion [43–45]. Taken together, these findings imply that diabetes leads to pan-coronary vulnerability and contributes to worse prognosis in CHD patients with diabetes.

### 4.3. Obesity and Coronary Atherosclerotic Plaque Vulnerability

Obesity is recognized as a traditional risk factor for CHD. An early IVUS study showed that obese patient had larger plaque area and higher risk of plaque rupture compared to nonobese patients [46]. Moreover, the amount of visceral adipose tissue was associated with the amount of noncalcified plaques, as demonstrated using computed tomography (CT)-coronary angiography [47]. However, few studies have investigated the potential association between obesity and coronary atherosclerotic plaque vulnerability, particularly via OCT. In our study, higher BMI was independently associated with plaque rupture, but not TCFA, as determined by OCT. This finding is inconsistent with a previous study, which showed that obesity was significantly correlated with TCFA detected by OCT [43]. These inconsistencies may be explained by different patient characteristics. Collectively, these findings highlight the importance of weight loss in preventing cardiovascular adverse events.

### 4.4. Study Limitations

Our study has limitations that should be taken into consideration when interpreting the results. First, this was a retrospective observational study, and causative associations between diabetes, obesity, and coronary vulnerability could not be derived based on the results. Secondly, we did not include patients with STEMI, and therefore the association between diabetes, obesity, and coronary vulnerability should be evaluated in future studies. Thirdly, we only analyzed plaque composition at the site of target lesions; thus, the association between diabetes, obesity, and coronary vulnerability in nontarget lesions should also be determined in future studies. Finally, a lack of longitudinal follow-up data prohibited assessment of the clinical impact of OCT analysis on future events.

## 5. Conclusions

Serum fibrinogen was not associated with coronary vulnerability in our cohort, but diabetes, higher BMI, and ACS were independently associated with coronary vulnerability as detected by OCT.

## Figures and Tables

**Table 1:** Risk factors and biochemical indices of patients according to plaque vulnerability.

	Ruptured plaque group	Nonrupture with TCFA group	Nonrupture and non-TCFA group	t/*χ*^2^	P
Male	15 (88.2)	13 (86.7)	74 (60.7)	8.177	0.017

Age	58.94±10.23	55.33±9.60	56.39±12.07	0.448	0.640

Hypertension	10 (58.8)	9 (60.0)	62 (50.8)	0.749	0.688

Diabetes mellitus	10 (58.8)	8 (53.3)	24 (19.7)	15.730	<0.001

Current smoking	11 (64.7)	9 (60.0)	46 (37.7)	6.436	0.040

Current drinking	4 (23.5)	1 (6.7)	26 (21.3)	2.373	0.305

Family history	2 (11.8)	1 (6.7)	26 (21.3)	2.931	0.231

BMI	29.09±3.88	26.64±2.45	24.60±2.98	17.847	<0.001

LDL-c (mmol/l)	2.39±0.87	2.48±0.54	2.36±0.94	0.104	0.902

HDL-c (mmol/l)	0.90±0.20	1.00±0.22	1.03±0.27	2.170	0.118

ApoA1 (g/L)	1.00±0.12	1.10±0.19	1.11±0.20	2.173	0.117

ApoB (g/L)	0.78±0.28	0.83±0.19	0.8±0.52	0.033	0.968

TC (mmol/l)	3.61±0.98	3.96±0.66	3.74±1.23	0.340	0.712

TG (mmol/l)	2.08±1.02	2.26±1.33	1.94±1.61	0.299	0.742

Lp(a) (g/L)	277.22±177.78	191.92±176.26	256.05±234.49	0.641	0.543

HbA1c (%)	7.07±1.34	6.80±1.03	6.32±1.28	1.802	0.172

Uric acid (*μ*mmol/L)	348.79±76.98	341.39±80.28	335.41±98.44	0.163	0.850

Creatinine (*μ*mmol/L)	76.29±17.46	74.58±17.28	74.25±18.77	0.091	0.913

Carbamide (mmol/l)	5.95±1.79	4.98±1.43	5.56±1.61	1.455	0.237

eGFR	112.59±47.06	106.5±31.03	107.65±36.96	0.143	0.867

Fibrinogen (g/L)	3.71±0.54	3.27±0.40	3.56±1.06	0.840	0.434

FDP (*μ*g/L)	1.50 (1.28, 3.35)	1.00 (0.88, 1.40)	1.50 (1.00, 2.70)	5.249	0.072

TBil (mmol/l)	11.93±3.89	12.89±4.11	13.60±10.11	0.267	0.766

DBiL (mmol/l)	2.88±1.47	3.53±1.47	3.74±2.67	0.893	0.411

IBiL (mmol/l)	9.13±3.80	9.36±4.00	9.54±5.87	0.043	0.958

PLT ^^^(10^∧^9/L)	223.12±51.27	237.6±77.93	232.33±65.22	0.214	0.808

MPV (fL)	10.31±0.75	10.43±1.38	10.75±1.08	1.676	0.191

PCT (%)	0.23±0.05	0.24±0.07	0.25±0.06	0.526	0.592

PDW	13.02±3.96	14.28±3.39	14.80±2.76	2.768	0.066

RBC(10^∧12^/L)	4.77±0.46	4.8±0.36	4.76±0.49	0.060	0.941

HCT (%)	0.44±0.05	0.43±0.04	0.43±0.04	0.277	0.758

HGB (g/L)	144.35±16.82	142.87±11.77	142.39±15.62	0.123	0.885

Hs-CRP	2.43 (0.82, 3.95)	0.86 (0.27, 2.15)	1.46 (0.55, 8.32)	0.831	0.660

ACS	13 (76.5)	10 (66.7)	39 (32.0)	17.105	<0.001

Aspirin	11 (64.7)	11 (73.3)	91 (74.6)	0.709	0.701

Statins	11 (64.7)	13 (86.7)	94 (77.0)	2.194	0.334

*β*-Blockers	7 (41.2)	3 (20.0)	46 (37.7)	2.001	0.368

ACEI/ARB	6 (35.3)	6 (40.0)	46 (37.7)	0.076	0.963

CCB	5 (29.4)	5 (33.3)	29 (23.8)	0.782	0.676

Oral hypoglycemic drugs	4 (23.5)	3 (20.0)	21 (17.2)	0.416	0.812

Insulin	2 (11.8)	1 (6.7)	13 (10.7)	0.295	0.863

Values are presented as n (%), or mean ± SD; BMI, body mass index; TG, triglyceride; TC, total cholesterol; HDL-C, high-density lipoprotein cholesterol; LDL-C, low-density lipoprotein cholesterol; BPC-blood platelet count; MPV, mean platelet volume; PCT, thrombocytocrit; PDW, platelet distribution width; RBC, red blood cell; PLT, platelet; HCT, hematocrit; HGB, hemoglobin; TBil, total bilirubin; DBiL, direct bilirubin; IBiL, unconjugated bilirubin; SAP, stable angina pectoris; UAP, unstable angina pectoris; NSTEMI, non–ST-segment elevation myocardial infarction; Apo A1, Apo lipoprotein AI; Apo B, Apo lipoprotein B; Lp (a), Lipoprotein (a); FDP, fibrinogen degeneration products; Hs-CRP, high sensitivity C-reactive protein.

**Table 2:** Coronary angiographic findings and OCT characteristics according to plaque vulnerability.

	Ruptured plaque group	Nonrupture with TCFA group	Nonrupture and non-TCFA group	t/*χ*^2^	P
ACS-IFC (%)				2.271	0.321

No	15 (88.2)	11 (73.3)	108 (88.5)		

Yes	2 (11.8)	4 (26.7)	14 (11.5)		

Vasa vasorum				1.826	0.401

No	16 (94.1)	12 (80.0)	111 (91.0)		

Yes	1 (5.9)	3 (20.0)	11 (9.0)		

Thrombus				31.431	<0.001

No	4 (23.5)	10 (66.7)	107 (87.7)		

Yes	13 (76.5)	5 (33.3)	15 (12.3)		

Macrophage accumulation				32.148	<0.001

0	3 (17.6)	3 (20.0)	79 (64.8)		

1	7 (41.2)	7 (46.7)	25 (20.5)		

2	5 (29.4)	4 (26.7)	18 (14.8)		

3	1 (5.9)	1 (6.7)	0 (0.0)		

4	1 (5.9)	0 (0.0)	0 (0.0)		

MLA(mm^2^)	3.28±1.89	3.51±2.08	3.50±1.97	0.090	0.914

NLA(mm^2^)	11.60±3.73	10.78±3.03	10.19±3.01	1.777	0.173

Rate of stenosis	81.12±15.89	75.67±13.35	72.93±17.14	1.870	0.158

Calcified nodule				1.137	0.547

No	17 (100.0)	15 (100.0)	113 (92.6)		

Yes	0 (0.0)	0 (0.0)	9 (7.4)		

Target vessel				5.880	0.208

LAD	10 (58.8)	10 (66.7)	95 (77.9)		

LCX	2 (11.8)	3 (20.0)	6 (4.9)		

RCA	5 (29.4)	3 (13.3)	21 (17.2)		

Lesion length	8.45±4.07	10.29±3.92	9.64±3.39	1.204	0.303

Location of target plaque				0.804	1.000

Pro	11 (64.7)	10 (66.7)	80 (65.6)		

Mid	6 (35.3)	5 (33.3)	40 (32.8)		

Distal	0 (0.0)	0 (0.0)	2 (1.6)		

Values are presented as n (%), or mean ± SD; ACS-IFC: Acute Coronary Syndrome with Intact Fibrous Cap; FCT, fibrous cap thickness; MLA, minimal lumen area; NLA, normal lumen area; Pro, proximal.

**Table 3:** Predictors of the presence of plaque vulnerability as detected by ruptured plaque or nonrupture with TCFA: results of multivariate logistic regression analysis.

Independent variable	Model 1			Model 2		
	P	OR	95% CI	P	OR	95% CI
Diabetes mellitus	0.036	4.703	1.106-19.989	0.022	4.450	1.242-15.939

Male	0.188	0.246	0.031-1.982	0.197	0.345	0.068-1.740

Current smoking	0.775	0.804	0.181-3.568	0.997	0.997	0.270-3.691

BMI	0.001	1.572	1.213-2.036	0.117	1.181	0.959-1.454

ACS	0.037	4.418	1.903-17.847	0.047	3.498	1.017-12.026

OR, odds ratio; CI, confidence interval.

**Table 4:** Fibrinogen levels in patients with different characteristics.

	Group	Fibrinogen	t/*χ*^2^	P
Gender	Female	3.61±1.12	1.436	0.153
	Male	3.42±0.54		

Age	<65y	3.56±1.04	0.104	0.917
	≥65y	3.54±0.79		

Hypertension	No	3.58±1.04	0.297	0.767
	Yes	3.53±0.91		

Diabetes mellitus	No	3.56±1.06	0.300	0.764
	Yes	3.51±0.69		

Current smoking	No	3.47±0.93	1.164	0.246
	Yes	3.65±1.01		

Current drinking	No	3.49±0.89	1.569	0.119
	Yes	3.79±1.22		

Family history of CAD	No	3.49±0.92	1.553	0.122
	Yes	3.80±1.16		

BMI	<24	3.53±1.07	0.033	0.968
	24-28	3.57±1.05		
	≥28	3.54±0.64		

HDL-c (mmol/l)	<1mmol/L	3.49±0.81	0.756	0.451
	≥1mmol/L	3.61±1.11		

LDL-c (mmol/l)	<3.1mmol/L	3.48±0.85	1.374	0.172
	≥3.1mmol/L	3.76±1.32		

T C (mmol/l)	<5.2mmol/L	3.50±0.85	0.786	0.448
	≥5.2mmol/L	3.91±1.79		

TG (mmol/l)	<2.3mmol/L	3.58±1.05	0.823	0.412
	≥2.3mmol/L	3.43±0.70		

Lp(a) (g/L)	<300mg/L	3.51±1.04	0.424	0.672
	≥300mg/L	3.59±0.76		

Clinical diagnosis	SAP	3.54±0.92	0.344	0.709
	UAP	3.63±1.15		
	NSTEMI	3.42±0.83		

Aspirin	Yes	3.75±1.15	1.397	0.165
	No	3.49±0.90		

Statins	Yes	3.72±1.09	1.310	0.192
	No	3.49±0.92		

*β*-Blockers	Yes	3.66±1.07	1.882	0.062
	No	3.36±0.75		

Abbreviations are the same as in Table 1.

**Table 5:** Coronary angiographic findings and OCT analysis in patients according to serum fibrinogen levels.

	Group	Fibrinogen<4.0	Fibrinogen>4.0	t/x^2^	P
FCT(*μ*m)		140 (60,230)	110 (30,200)	1.055	0.291
Lipid arc, degree		116 (0,174)	107 (0,178)	0.008	0.994
Rupture (%)	No	117 (90.0)	20 (83.3)	0.364	0.546
	Yes	13 (10.0)	4 (16.7)		
ACS-IFC (%)	No	116 (89.2)	18 (75.0)	2.481	0.115
	Yes	14 (10.8)	6 (25.0)		
Macrophage accumulation	0	71 (54.6)	14 (58.3)	4.744	0.303
	1	36(27.7)	3 (12.5)		
	2	20 (15.4)	7 (29.2)		
	3	2 (1.5)	0 (0.0)		
	4	1 (0.8)	0 (0.0)		
Vasa vasorum	No	117 (90.0)	22 (91.7)	0.000	1.000
	Yes	13 (10.0)	2 (8.3)		
Thrombus	No	104 (80.0)	17 (70.8)	1.011	0.315
	Yes	26 (20.0)	7 (29.2)		
Diameter stenosis, %		74.43±17.17	72.29±14.74	0.572	0.568
Calcified nodule	No	123 (94.6)	22 (91.7)	0.009	0.926
	Yes	7 (5.4)	2 (8.3)		
TCFA		25 (19.2)	5 (20.8)	0.000	1.000
Minimal lumen area (mm^2^)		3.57±2.03	2.92±1.46	1.511	0.133
Normal lumen area (mm^2^)		10.60±3.13	9.90±3.30	1.000	0.319
Lesion Length		9.74±3.61	8.64±2.90	1.413	0.160
Characteristic of plaque	Lipid	84 (64.6)	15 (62.5)	0.042	0.979
	Calcified	20 (15.4)	4 (16.7)		
	Fibrotic	26 (20.0)	5 (20.8)		
Target vessel	LAD, n (%)	98 (75.4)	17 (70.8)	3.436	0.179
	LCX, n (%)	7 (5.4)	4 (16.7)		
	RCA, n (%)	25 (19.2)	3 (12.5)		
Location of target plaque	Proximal	89 (68.5)	12 (50.0)	3.590	0.155
	Mid	39 (30.0)	12 (50.0)		
	Distal	2 (1.5)	0 (0.0)		

Abbreviations are the same as in Table 2.

## Data Availability

We collected the demographic data, clinical characteristics, risk factors, blood samples, biochemical data, data of ECG, echocardiography, coronary angiography, and optical coherence tomography images in the First Affiliated Hospital of Xinjiang Medical University from January 2015 to August 2018. The data that support the findings of this study are available from the First Affiliated Hospital of Xinjiang Medical University, but restrictions apply to the availability of these data, which were used under license for the current study, and so are not publicly available. Data are however available from the authors upon reasonable request and with permission of the First Affiliated Hospital of Xinjiang Medical University.
